# Associations of maternal, perinatal and postnatal factors with the eruption timing of the first primary tooth

**DOI:** 10.1038/s41598-019-39572-w

**Published:** 2019-02-25

**Authors:** Huaying Wu, Ting Chen, Qian Ma, Xiangqin Xu, Kaipeng Xie, Yaming Chen

**Affiliations:** 1 Department of Polyclinics, The Affiliated Stomatological Hospital of Nanjing Medical University, Nanjing, 210029 China; 2 Department of Stomatology, The Affiliated Obstetrics and Gynecology Hospital of Nanjing Medical University, Nanjing Maternity and Child Health Care Hospital, Nanjing, 210004 China; 3 Nanjing Maternity and Child Health Care Institute, The Affiliated Obstetrics and Gynecology Hospital of Nanjing Medical University, Nanjing Maternity and Child Health Care Hospital, Nanjing, 210004 China; 4 Department of Women Health Care, The Affiliated Obstetrics and Gynecology Hospital of Nanjing Medical University, Nanjing Maternity and Child Health Care Hospital, Nanjing, 210004 China; 5 State key Laboratory of Reproductive Medicine, The Affiliated Obstetrics and Gynecology Hospital of Nanjing Medical University, Nanjing Maternity and Child Health Care Hospital, Nanjing, 210004 China

## Abstract

We recruited 1296 mothers in their first trimester from the Affiliated Obstetrics and Gynecology Hospital of Nanjing Medical University between May 2014 and September 2015 to investigate the associations of maternal, perinatal and postnatal factors with the eruption timing of the first primary tooth (ETFPT) in a Chinese population. We collected maternal demographic information and clinical data during the perinatal and postnatal period, and oral examinations of the infants were performed by a doctor at 6, 9 and 12 months of age. Multiple regression analysis was used to identify significant explanatory variables for ETFPT. The mean age at eruption of the first primary tooth for all the infants was 6.82 ± 1.90 months. After adjustment for confounders, higher maternal childbearing age (β = 0.57; 95%CI = 0.13–1.02), female sex (β = 0.26; 95%CI = 0.07–0.52), and low birth weight (β = 0.98; 95%CI = 0.20–1.76) were significantly associated with delayed eruption of the first primary tooth, while macrosomia (β = −0.79; 95%CI = −1.30–−0.28) was significantly associated with earlier eruption of the first primary tooth. Maternal childbearing age, infant sex and infant birth weight were significant determinants of ETFPT.

## Introduction

The formation and development of human primary teeth begins at the end of the fifth week of gestation^1^. Teeth are formed in the upper and lower jaw through mutual, subtle and sophisticated interactions between the dental epithelium and oral ectomesenchyme involving the expression of several tooth-related genes^1^. Primary tooth eruption is a complex and highly regulated process in which teeth enter the mouth and become visible during a certain time period. In most infants, the first primary tooth to erupt is the central mandibular incisor, which erupts between 2 and 15 months of age^2^. The complete primary dentition erupts from 10 to 33 months depending on the position and type of tooth^3^.

Variations in the eruption timing of the first primary tooth (ETFPT) are considered multifactorial. Eruption is under strong genetic control, and the estimates of narrow-sense heritability are over 70%^4^. Furthermore, genome-wide association studies (GWAS) have identified some candidate genes associated with tooth development, such as *KCNJ2*, *EDA*, *HOXB2*, *RAD51L1*, *IGF2BP1*, *HMGA2* and *MSRB3*^5^. There are ethnicity- and sex-related differences in the timing of primary tooth eruption^6^. However, external environmental factors also make significant contributions to the timing of the primary tooth eruption. Maternal exposure to tobacco during pregnancy^7,8^, infant birth weight^9,10^, birth length^11^, nutritional state at birth and at postnatal timepoints^12^, gestational age^13^, method of infant feeding^8,14^ and socioeconomic situation^15^ have been reported to be significant determinants of the eruption of primary teeth. Delayed tooth eruption has been reported in premature infants^13,16^ with small gestational age and low birth weight and in those with systemic disorders, such as hypothyroidism^17^, while accelerated tooth eruption has been observed in children whose mothers smoked during pregnancy^7,8^ as well as in those with childhood obesity^18^ and diabetes mellitus^19^.

Although several studies have examined the factors influencing tooth eruption, their results are inconsistent^8,14,20–22^. Most of the studies focused only on factors within a defined period of time and based their findings on evidence from small cohorts, while some studies focused on the whole pregnancy. A prospective cohort study in Turkey^23^ found that growth parameters and feeding patterns may be determinants of the timing of tooth eruption. The GUSTO cohort study in Singapore^24^ found that infant weight gain from birth to 3 months, ethnicity and maternal childbearing age were associated with the timing of the eruption of the first tooth. A study from the Southampton Women’s Survey^25^ found that maternal smoking and socioeconomic status were associated with tooth eruption. Taken together, these cohort studies^23–25^ were based on relatively large populations and focused on comprehensive data, including maternal, perinatal and postnatal data; in contrast, their results are not entirely consistent.

There is definite evidence that children in different geographic regions have different eruption timing of the primary teeth^26,27^. Importantly, other factors, including socioeconomic factors, nutritional status, maternal educational levels and overall maternal health vary from country to country^14,28^. In the United States, primary tooth eruption timing differs among American Indian, Black and White children^29^. The GUSTO cohort study in Singapore^24^ found that compared to Chinese children, Malay and Indian children experienced significantly delayed tooth eruption. Meanwhile, primary tooth eruption timing differs between Indian children in Singapore^24^ and in India^30,31^. However, there is a lack of population data for residents in Nanjing of China. Therefore, our study aimed to evaluate the relationships between maternal, perinatal and postnatal factors and ETFPT of infants in a large cohort of Chinese mothers in Nanjing, China.

## Results

### Subject characteristics

The demographic and clinical characteristics of 1109 mother-child pairs are shown in Table 1. The maternal childbearing age ranged from 20 to 41 years. Among the mothers, 84 (7.57%) were over 35 years of age, and 50 (4.51%) gave birth at a gestational age of less than 37 weeks. Among the infants, 524 (47.20%) were females, 26 (2.34%) were classified as low birth weight (<2500 g) and 58 (5.23%) had macrosomia (>4000 g).

**Table 1:** Demographic characteristics, ETFPT, and the significance of their association in 1109 mother-child pairs.

Factors	N (%)	ETFPT (months)				*P* ^*a*^	*P* ^*b*^
		Mean	SD	Minimum	Maximum		
	Total = 1109	6.82	1.90	3.0	13.0		
Mother’s childbearing age (years)							
<25	41 (3.70%)	6.90	2.51	4.0	12.0	0.755	0.452
25–35	984 (88.73%)	6.78	1.87	3.0	13.0	—	—
>35	84 (7.57%)	7.31	1.92	4.0	13.0	**0.014**	**0.014**
Exposure to secondhand smoke							
Yes	150 (13.53%)	7.01	1.95	3.0	13.0	0.192	0.261
No	959 (86.47%)	6.80	1.89	3.0	13.0	—	—
Prepregnancy BMI (kg/m^2^)							
<18.5	102 (9.20%)	6.86	1.98	3.0	13.0	0.846	0.924
18.5–23.9	929 (83.77%)	6.82	1.89	3.0	13.0	—	—
24.0–27.9	66 (5.95%)	6.76	1.98	4.0	11.0	0.809	0.620
≥28.0	12 (1.08%)	7.21	1.97	4.0	10.0	0.480	0.364
Parity							
Multiparous	183 (16.50%)	6.71	1.71	3.0	11.0	0.342	0.607
Primiparous	926 (83.50%)	6.85	1.94	3.0	13.0	—	—
Gestational age (weeks)							
<37	50 (4.51%)	7.39	1.78	4.0	13.0	**0.032**	**0.021**
≥37	1059 (95.49%)	6.80	1.90	3.0	13.0	—	—
Mode of delivery							
Caesarean	431 (38.86%)	6.82	1.94	3.0	13.0	0.919	0.668
Vaginal	678 (61.14%)	6.83	1.88	3.0	13.0	—	—
Infant sex							
Female	524 (47.20%)	6.98	1.91	3.0	13.0	**0.009**	**0.007**
Male	585 (52.80%)	6.68	1.89	3.0	13.0	—	—
Birth weight (g)							
<2500	26 (2.34%)	8.23	1.68	5.5	11.0	<**0.001**	<**0.001**
2500–4000	1025 (92.43%)	6.84	1.89	3.0	13.0	—	—
>4000	58 (5.23%)	5.91	1.70	3.0	11.0	<**0.001**	<**0.001**
Feeding model							
Breast feeding	207 (18.67%)	6.97	1.92	3.0	13.0	0.236	0.154
Artificial feeding	40 (3.61%)	6.68	1.76	3.0	10.5	0.699	0.801
Mixed feeding	862 (77.73%)	6.80	1.90	3.0	13.0	—	—
Breast feeding duration (months)							
<6	90 (8.12%)	6.98	2.10	3.0	13.0	0.423	0.633
≥6	1019 (91.88%)	6.81	1.88	3.0	13.0	—	—

^a^*p* values were determined by Student’s t-tests compared to the reference group.

^b^*p* values were determined by Mann-Whitney U-test compared to the reference group.

*Abbreviations: ETFPT is eruption timing of the first primary tooth, N is sample size, SD is standard deviation, kg is kilograms, m is meters, g is grams.

### ETFPT

**Figure 1:**
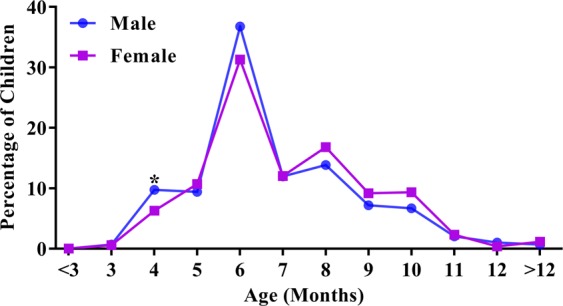
Distribution of 1109 children according to the eruption timing of the first primary tooth (males, females). *P < 0.05.

**Figure 2:**
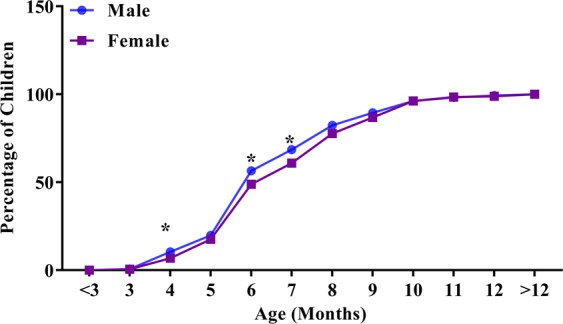
Cumulative distribution of 1109 children according to the eruption timing of the first primary tooth (males, females) *P < 0.05.

The mean ETFPT of all the infants was 6.82 ± 1.90 months. As shown in Table 1, ETFPT was significantly delayed in infants born to mothers older than 35 years, infants with a low gestational age and infants with low birth weight, while the ETFPT was earlier in male infants and those with macrosomia (all *p* < 0.05). Interestingly, the first eruption peak for both females and males was 6 months, and the second peak was 8 months (Fig. 1). Within the first 7 months of life, 68.55% of the males had erupted their first primary tooth, compared with only 60.88% of the females (*p* = 0.008) (Fig. 2).

### Potential determinants of ETFPT

Several factors showed significant associations with ETFPT in the univariate analysis, including maternal factors, such as increased maternal childbearing age and low gestational age, and perinatal and postnatal factors, such as female sex, low birth weight and macrosomia (all *p* < 0.05, Table 2). No significant associations with ETFPT were observed for other maternal, perinatal and postnatal factors, including exposure to secondhand smoke during pregnancy, prepregnancy body mass index (BMI), feeding model or breast feeding duration. The multivariate analysis indicated that the significant determinants of ETFPT were maternal childbearing age and infant sex and birth weight. Mothers of higher maternal childbearing age (β = 0.57, 95%CI = 0.13–1.02, *p* = 0.010), female infants (β = 0.26, 95%CI = 0.07–0.52, *p* = 0.022), and low infant birth weight (β = 0.98, 95%CI = 0.20–1.76, *p* = 0.010) were significantly associated with delayed ETFPT, while macrosomia (β = −0.79, 95%CI = −1.30–−0.28, *p* = 0.000) was significantly associated with earlier ETFPT (Table 2).

**Table 2:** Association of maternal, perinatal and postnatal information factors with ETFPT.

Factors	Univariate analysis		Multivariable analysis^b^	
	β (95%CI)	*P* value	β (95%CI)	*P* value
Mother’s childbearing age (years)	0.00 (−0.03,0.04)	0.845	0.00 (−0.03, 0.04)	0.910
<25	0.12 (−0.47, 0.72)	0.681	0.15 (−0.44, 0.74)	0.620
25–35^a^	—	—	—	—
>35	0.53 (0.10, 0.95)	**0.015**	0.57 (0.13, 1.02)	**0.010**
Exposure to secondhand smoke				
Yes	0.22 (−0.11, 0.55)	0.192	0.25 (−0.07, 0.58)	0.130
No^a^	—	—	—	—
Prepregnancy BMI (kg/m^2^)				
<18.5	0.04 (−0.35, 0.43)	0.847	−0.02 (−0.41, 0.36)	0.900
18.5–23.9	—	—	—	—
24.0–27.9	−0.06 (−0.53, 0.42)	0.810	0.18 (−0.30, 0.66)	0.460
≥28.0^a^	0.39 (−0.53, 0.42)	0.483	0.32 (−0.75, 1.39)	0.560
Parity				
Multiparous	−0.13 (−0.44, 0.17)	0.381	−0.16 (−0.49, 0.17)	0.340
Primiparous^a^				
Gestational age (weeks)				
<37	0.59 (0.05, 1.13)	**0.032**	0.01 (−0.57, 0.59)	0.970
≥37^a^	—	—	—	—
Mode of delivery				
Caesarean	−0.01 (−0.24, 0.22)	0.919	0.00 (−0.23, 0.24)	0.980
Vaginal^a^	—	—	—	—
Infant sex				
Female	0.30 (0.07, 0.52)	**0.009**	0.26 (0.07, 0.52)	**0.022**
Male^a^	—	—	—	—
Birth weight (g)				
<2500	1.39 (0.65–2.12)	<**0.001**	0.98 (0.20, 1.76)	**0.010**
2500–4000^a^	—	—	—	—
>4000	−0.93 (−1.43, −0.44)	<**0.001**	−0.79 (−1.30, −0.28)	**0.000**
Feeding model				
Breast feeding	0.18 (−0.11, 0.46)	0.234	0.17 (−0.12, 0.46)	0.250
Artificial feeding	−0.12 (−0.72, 0.48)	0.700	−0.18 (−0.88, 0.52)	0.610
Mixed feeding^a^	—	—	—	—
Breast feeding duration (months)				
<6	0.17 (−0.24, 0.58)	0.423	0.08 (−0.32, 0.49)	0.690
≥6^a^	—	—	—	—

^a^Reference group.

Bold values indicate significant results.

^b^Adjusted for all factors except the investigated factor.

*Abbreviations: ETFPT is eruption timing of the first primary tooth, CI is confidence interval, kg is kilograms, m is meters, g is grams.

## Discussion

In this prospective study, we systematically analyzed the association of maternal, perinatal and postnatal factors with the ETFPT. We observed that the first eruption peak for all infants was in the 6th month, and the second peak was in the 8th month. However, the ETFPT was earlier in male infants than in females. Subsequently, we found that higher maternal childbearing age and low birth weight were significantly associated with delayed ETFPT, while macrosomia was significantly associated with earlier ETFPT.

Comparing our study with the GUSTO study^24^ revealed some differences in results. First, we found substantial differences (*p* < 0.001) in the ETFPT between Chinese children in Nanjing, China, and Singapore. The mean ETFPT of all the infants in our study was 6.82 ± 1.90 months, while in the GUSTO study in Singapore, the mean ETFPT of the Chinese children was 7.80 ± 2.20 months. Similarly, the mean ETFPT of Indian children was 8.15 ± 1.69 months in a major city in North India^30^ and 9.50 ± 2.70 months in Singapore^24^. Meanwhile, another study in India reported that the mean ETFPT of Indian children in Bhopal was 11.40 ± 3.43 months^31^. Although differences in tooth eruption timing between shared ancestry populations in different countries/communities do exist, the reasons may include nutrition, socioeconomic status, climate, and environmental factors, such as the fluoride content in drinking water^30^. Common environmental toxins can also affect tooth development in human embryos^32^. However, for any one factor, the results are inconsistent and inconclusive. The tooth eruption timing reported in this study differs from those reported in longitudinal studies in other countries^33,34^, suggesting that population-level differences in the ETFPT may exist. The ETFPT is ethnicity and community dependent. Since the current standards of primary teeth eruption timing are based mainly on Western populations, our data can be used as a reference for future clinical studies in Nanjing of China.

Furthermore, our study showed that maternal childbearing age as a continuous variable have no statistics significance, however as a ordinal variable that increasing maternal childbearing age is associated with delayed ETFPT in infants. This finding disagrees with the results of the GUSTO study in Singapore^24^, which previously uncovered an association between higher maternal childbearing age and earlier ETFPT in infants. Previous work has demonstrated a similar relationship between maternal childbearing age and child growth parameters in which increased maternal childbearing age was associated with taller stature in children - on average, children of mothers who were older than 30 years at childbirth were 1.5 cm taller than those of mothers who were younger than 30 years^35^. The mechanisms underlying these differences are unclear but may include ethnic or population history differences and environmental and/or genetic factors. Additionally, the literature suggests that factors such as ethnicity or population history may influence tooth formation and eruption^36^.

The GUSTO study in Singapore^24^ did not find a sex-related difference; however, in the present study, we found a sex difference in the ETFPT, which was earlier in males. Our results align with previous studies that found that the primary teeth erupt earlier in males than in females^37–40^. The reason for the differences in the timing of tooth eruption between males and females is poorly understood. We hypothesize that the earlier onset of the primary dentition is related to differences in sexual maturity and may be partially attributed to environmental and/or genetic influences on growth^41^.

Previous studies have also suggested that the timing of primary tooth eruption is significantly related to general somatic growth and nutritional status^42^. Birth weight has been used as a marker of intrauterine nutritional environment, with low birth weight indicating poor fetal nutrition. Additionally, children who have experienced nutritional deficiencies show delayed primary teeth eruption^43^. Interestingly, we found that the first primary tooth eruption occurred earlier in infants with macrosomia, while it was delayed in infants with low birth weight; these findings are consistent with studies that focused on other populations^44,45^. Even when chronological age was adjusted for prematurity, infants with low birth weight have been shown to have greater likelihood of delayed ETFPT^46^.

According to previous research, there was an association between the earlier eruption of permanent teeth and childhood obesity^18^. Similarly, the GUSTO study in Singapore^24^ reported that earlier tooth eruption was associated with infant weight gain from birth to 3 months. Additionally, our study demonstrated that macrosomia is associated with earlier eruption of the primary teeth. Because birth weight is related to pregnancy and perinatal nutritional status and conditions, the findings of this study also suggest that primary tooth eruption may be an indicator of the nutritional status of the mother during pregnancy. As a result, we proposed that adequate nutrition during pregnancy and early life may prevent delayed ETFPT in infants.

Previous studies have also found that children born to mothers who smoked during pregnancy had an earlier ETFPT^7^. Reports indicate that only 2.4% of the women in China are tobacco smokers^47^, and most women who do smoke quit after they became pregnant^48^. Few mothers in China actively smoke during pregnancy, but secondhand smoke exposure still exists. However, we did not find an association between ETFPT and the mother’s exposure to secondhand smoke during pregnancy. Additionally, some previous studies have noted an association between breast feeding and the timing of the eruption of primary teeth, finding that children who were not breast fed had delayed tooth eruption^49^. The effect of breast feeding was also examined in this study, but we found that neither the breast feeding model nor the duration of breast feeding were significantly related to the ETFPT, which agreed with another report^50^.

Our study has a number of strengths. First, our participants were drawn from a systematic screening of pregnant women in a population-based, large study performed in Nanjing, China. Moreover, the relatively large sample size in this study provided good statistical power. There are also several limitations to this study. First, it used information from a cohort based on only one hospital in one city. The annual delivery rate of Nanjing babies in this hospital was about 20%. Therefore, the results should be considered with caution. Second, some reported risk factors for ETFPT, such as sociodemographic and socioeconomic characteristics, were not considered for adjustment in this study due to the high percentage of missing data. Third, the data on the timing of tooth eruption were reported by the mothers and are thus subject to error. To further explore the link between primary tooth eruption and other postnatal factors, further studies are ongoing. In particular, future studies will explore the association between the eruption timing of primary teeth and the development of subsequent dental caries, which has received little attention in the literature.

## Conclusions

Based on this study’s findings, the following conclusions can be drawn:

1. Higher maternal childbearing age, female sex in infants and low infant birth weight were significantly associated with delayed ETFPT, while macrosomia was significantly associated with earlier ETFPT.
2. Considering the limitations of this study, no associations were observed between other maternal, perinatal or postnatal factors and ETFPT.
3. The ETFPT differs depending on infants’ ethnicity and community, likely in response to variation in environmental, developmental and genetic factors.

## Methods

### Study design

This study was conducted according to the guidelines in the Declaration of Helsinki, and all procedures involving human subjects were approved by the Institutional Review Board of Nanjing Maternity and Child Health Care Institute. A total of 1296 singleton pregnant women in their first trimester were recruited between May 2014 and September 2015. The inclusion criteria included women who intended to give birth at the Affiliated Obstetrics and Gynecology Hospital of Nanjing Medical University and planned to reside in Nanjing, China, for the 5 years after being recruited into this study. The exclusion criteria included women who had chronic diseases requiring medication (e.g., prediagnosed diabetes) and those who abused alcohol or substances. All participants signed written free informed consent forms for themselves and their children.

### Maternal questionnaires and clinical data

Maternal general demographic information regarding childbearing age, prepregnancy BMI, pregnancy tobacco exposure, parity, personal health and family history of disease were collected from a detailed questionnaire. The clinical data of the women during pregnancy, including gestational age and delivery method, were extracted from hospital laboratory records.

The maternal childbearing age of the participants was divided into three groups: <25 years, 25–35 years, >35 years. BMI was calculated as weight (kg)/height (m)^2^. The World Health Organization (WHO) criteria were used to classify the women’s BMIs into four groups: underweight (<18.5 kg/m^2^), normal (18.5–23.9 kg/m^2^), overweight (24–27.9 kg/m^2^), and obese (>28.0 kg/m^2^). The infants’ gestational age at birth was divided into two groups: low gestational age, which was less than 37 weeks, and normal gestational age, which was at least 37 weeks.

Perinatal details, such as infant sex and birth weight, were also obtained. Infant birth weight was divided into three groups: low birth weight (<2500 g), normal birth weight (2500–4000 g), and macrosomia (>4000 g).

### Child questionnaires and oral examinations

Questionnaires regarding child factors, including feeding mode (breast feeding, artificial feeding or mixed feeding) and duration of breast feeding were collected at subsequent return visits. All oral examinations of the infants were performed at 6, 9, and 12 months of age by one experienced senior dentist. Meanwhile, a licensed dental assistant checked and recorded the data for each child. Training and calibration sessions were done before the oral examinations. Kappa was used to assess the intra-examiner reliability and the inter-examiner reliability. To this end, test-retest was done in first 20 samples for the observers and in 50 cases selected randomly, oral examinations were completed by the two examiners. The agreement between test and retest was 100% and the agreement between the dentist and the dental assistant was also excellent (Kappa = 1). The examinations were performed under natural light in a dental chair. At each visit, the mothers were asked the age or date at which the child’s first primary tooth erupted. The aim of the oral examinations was to ascertain the eruption of the child’s first primary tooth and its eruption mode. The eruption of a tooth was defined as the appearance of any part of the tooth piercing the gum by using Federation Dentaire Internationale (FDI) standards^51^.

**Figure 3:**
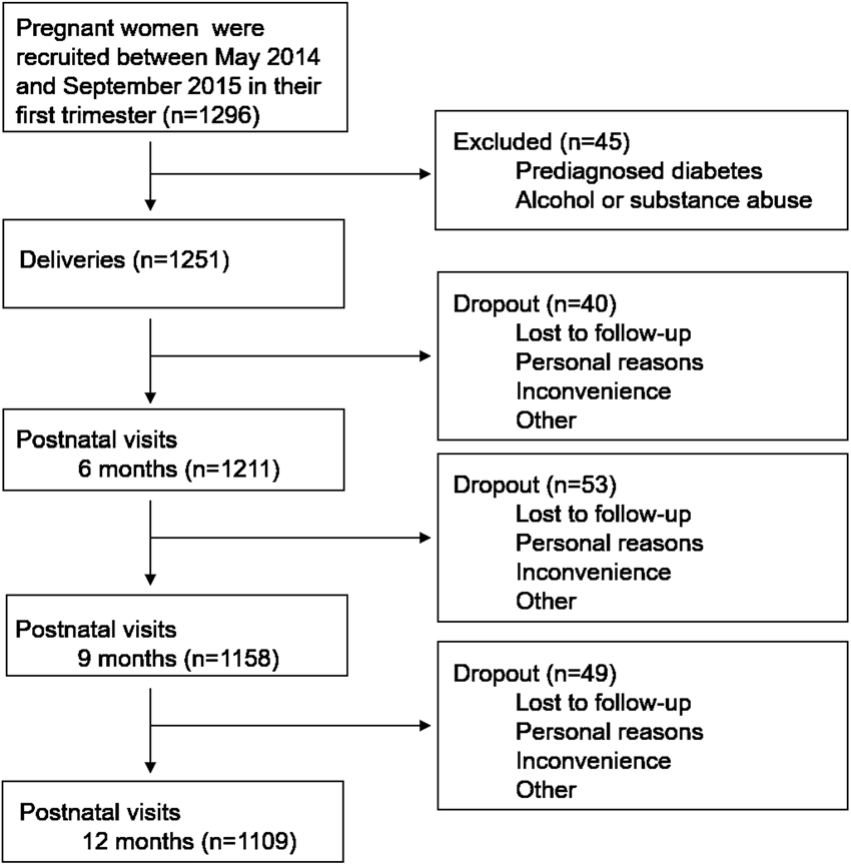
Recruited and follow-up process of the study.

Of the total 1296 singleton pregnant women, 45 were excluded because of prediagnosed diabetes, alcohol or substance abuse, and 1251 live-born infants were delivered. During the 6-, 9-, and 12-month return visits, 40, 53 and 49 patients dropped out because of loss to follow-up, personal reasons, inconvenience and other factors. After 12 months of oral examinations, sufficient information was available for 1109 mother-child pairs to perform further analysis (Fig. 3).

### Statistical analysis

Maternal, perinatal and postnatal information was collected to identify potential determinants of ETFPT (Table 3). Descriptive statistics, including means ± standard deviations, minimums, maximums and frequencies (percentages) were calculated for all variables. For children with no tooth eruption at the age of one year, the maximum value was recorded as 13 months because the 12-month visit occurred when the child between 11 to 13 months old. Student’s t-tests and Mann-Whitney U-tests were used to examine the difference in the ETFPT between groups. Chi-squared and Fisher exact tests were used to examine differences between percentage of children and ETFPT group. Univariate and multivariate linear regression models were used to identify the significant determinants linked to ETFPT. Statistical analyses were performed with R software (version 3.2.5), and *p* < 0.05 was considered statistically significant.

**Table 3:** Factors tested for association with ETFPT*.

Potential factors	Description
Maternal factors	Maternal childbearing age (years): (1) < 25; (2) 25–35; (3) > 35
	Maternal prepregnancy BMI: (1) Underweight (<18.5 kg/m^2^); (2) Normal (18.5–23.9 kg/m^2^); (3) Overweight (24–27.9 kg/m^2^); (4) Obese (>28.0 kg/m^2^)
	Exposure to secondhand smoke during pregnancy:(1) Yes; (2) No
	Parity:(1) Multiparous; (2) Primiparous
	Gestational age (weeks): (1) < 37; (2) ≥ 37
	Mode of delivery: (1) Caesarean; (2) Vaginal
Perinatal factors	Birth weight (g): (1) < 2500; (2) 2500–4000; (3) > 4000
	Child’s sex: (1) Male; (2) Female
Postnatal factors	Feeding model: (1) Breast feeding; (2) Artificial feeding; (3) Mixed feeding
	Duration of breast feeding (months): (1) < 6; (2) ≥ 6

*Abbreviations: ETFPT is eruption timing of the first primary tooth, kg is kilograms, m is meters, g is grams.
